# Analysis of the Bioactive Compounds and Physiological Activities of Commonly Consumed Noni Juice in Republic of Korea

**DOI:** 10.3390/foods14213732

**Published:** 2025-10-30

**Authors:** Xiaolu Fu, Min-Hye Kim, Geon Oh, Ji-Hyun Im, June-Seok Lim, Yeon-Seok Seong, Jae-Yeon Lee, Eun Young Park, Do Sang Lee, Im-Joung La, Ok-Hwan Lee

**Affiliations:** 1 Department of Food Biotechnology and Environmental Science, Kangwon National University, Chuncheon 24341, Republic of Korea; fuxiaolu2019@gmail.com (X.F.); minhye8733@naver.com (M.-H.K.); gun0195@kangwon.ac.kr (G.O.); ijh108020@gmail.com (J.-H.I.); dlawnstjr725@naver.com (J.-S.L.); seoksk59@kangwon.ac.kr (Y.-S.S.); 2 NSTBIO Co., Ltd., Incheon 21984, Republic of Korea; jyleebio@nstbio.co.kr (J.-Y.L.); pey@nstbio.co.kr (E.Y.P.); 3 Atomy R&D Center, Gongju 32511, Republic of Korea; ldosang@atomyorot.kr (D.S.L.)

**Keywords:** noni (*Morinda citrifolia* L.), noni juice, bioactive compounds, physiological activities

## Abstract

Noni (*Morinda citrifolia* L.) juice is increasingly recognized for its potential health-promoting properties. In this research, the bioactive compounds and physiological effects of commercial noni juice products in Korea were assessed. Noni juice was found to contain high levels of total phenolics (6.39 ± 1.45 mg gallic acid equivalents (GAE)/g) and proanthocyanidins (8.64 ± 6.20 mg catechin equivalents (CE)/g). Furthermore, it exhibited potent antioxidant activities, with 1,1-diphenyl-2-picrylhydrazyl (DPPH) and 2,2′-azino-bis(3-ethylbenzothiazoline-6-sulfonic acid) diammonium salt (ABTS) radical scavenging activities of 44.03 ± 14.88% and 55.91 ± 2.62%, respectively, which exceeded those reported for common fruit juices such as apple, orange, and blueberry. Additionally, noni juice reduced lipid accumulation by 5.92% and reactive oxygen species (ROS) levels by 7.23% in 3T3-L1 adipocytes; improved fusion index to 81.44% and restored myotube diameter by 37.24% in dexamethasone-induced C2C12 cells; and suppressed LPS-induced nitric oxide (NO) production. These results suggested that noni juice has anti-inflammatory, anti-obesity, anti-muscle atrophy, and antioxidant properties, supporting its potential as a functional health beverage.

## 1. Introduction

Noni (*Morinda citrifolia* L.) is a fruit widely distributed in the South Pacific and other tropical regions, and its use in traditional medicine dates back over 2000 years [1]. Traditionally, noni fruit has been used to treat inflammation, infections, arthritis, fever, and digestive disorders [2]. Numerous studies have reported that noni fruits contain a variety of bioactive compounds, such as coumarins, flavonoids, lignans, phenolics, iridoids, and amino acids, which are believed to play significant roles in the prevention or treatment of various diseases [3,4,5]. The main iridoid compounds found in noni fruit are deacetylasperulosidic acid (DAA), asperuloside (Asp), and asperulosidic acid (AA) [6,7]. Scopoletin (Sco), a coumarin derivative, is also known to be a representative bioactive component of the noni fruit [8]. Furthermore, Mani et al. [9] confirmed that noni fruit possesses diverse health-enhancing properties, including antioxidant, anticancer, anti-inflammatory, and antidiabetic effects. However, because of the presence of high concentrations of hexanoic acid, octanoic acid, and their methyl esters, noni fruit emits an unpleasant smell; moreover, its poor taste means that it is rarely eaten fresh and is typically processed into juice, powder, or capsules [10,11]. Moreover, further studies have indicated that noni juice products derived from processed noni fruits demonstrate superior antioxidant, immunomodulatory, anticancer, and anti-inflammatory properties than the raw fruit itself [12,13].

While noni juice is typically produced by pressing fresh noni fruits, it can also be made by adding other fruit extracts to enhance the flavor and aroma [14]. Noni juice has been commercially available in the USA since 1990. Subsequently, in 2003, the European Commission authorized its commercialization in Europe as an innovative food product, and since then has become popular worldwide [15]. In addition, fermentation processing has been widely applied to improve palatability and enhance the functional properties of noni juice. Among the various noni-based products, fermented noni juice has attracted increasing attention [16]. It is produced through a fermentation process using endogenous enzymes and natural symbiotic microorganisms, and is considered a novel, highly nutritious beverage [14,17,18]. Fermented noni juice exhibits significantly improved antioxidant activity compared to unfermented noni juice [19]. It is important to note that most commercial noni juice products contain other fruit concentrates or additives to improve taste and stability. Although these ingredients may contribute to the observed biological activities, previous studies have reported that noni juice exhibits superior antioxidant capacity compared with most common fruit juices, such as blueberry [20]. Therefore, it is plausible that noni may play a major role in the observed bioactivities of these commercial products. However, potential synergistic effects among mixed fruit components cannot be excluded. Additionally, a study by West [12] suggested that regular and sustained consumption of noni juice may provide significant health benefits. Currently, commercial noni juice is widely marketed as a dietary supplement and functional beverage, and plays a particularly important role in the functional food market [21].

Although some studies have explored the various health benefits of noni juice and fermented noni juice [2,13], comprehensive evaluations of the bioactive compounds and physiological activities of the fermented noni juice products commonly available in the Korean market remain limited. Therefore, this study aimed to integrate and average the analytical data from six commercial noni juice products, including both fermented and unfermented types, by combining high-performance liquid chromatography (HPLC) in conjunction with a photodiode array (PDA) detector, and multiple in vitro cellular assays. The results were subsequently compared with reported data on other commercial fruit juices to assess the relative functional potential of noni juice. This comparative approach provides a scientific basis for the health-promoting properties of noni juice and further underscores its unique potential and application value in the health-functional beverage market.

## 2. Materials and Methods

### 2.1. Chemicals

Scopolin (Scol) (CAS No. 531-44-2), Sco (CAS No. 92-61-5), DAA (CAS No. 14259-55-3), Asp (CAS No. 14259-45-1), and AA (CAS No. 25368-11-0) were purchased from ChemFaces (Wuhan, China). Formic acid (purity ≥ 98%) was obtained from Junsei (Tokyo, Japan) and subsequently diluted in distilled water (DW) to prepare a 0.1% mobile phase. Acetonitrile (ACN; purity ≥ 99%) was sourced from J.T. Baker (Philipsburg, NJ, USA). 1,1-diphenyl-2-picrylhydrazyl (DPPH), aluminum chloride, quercetin, Folin–Ciocalteu’s reagent, potassium acetate, sodium carbonate, gallic acid, fluorescein sodium salt (FL), potassium persulfate, ferric chloride (FeCl_3_·6H_2_O), trichloroacetic acid (TCA), 2,2′-azino-bis (3-ethylbenzothiazoline-6-sulfonic acid) diammonium salt (ABTS), 2,4,6-tripyridyl-s-triazine (TPTZ), 2,2-azobis(2-amidino propane) dihydrochloride (AAPH), vanillin, (+)-catechin, lipopolysaccharides (LPS), potassium ferricyanide, sulfanilamide, sodium nitrite (NaNO_2_), 3-isobutyl-1-methylxanthine (IBMX), dexamethasone (DEX), 6-hydroxy-2,5,7,8-tetramethylchroman-2-carboxylic acid (Trolox), bisphenol A(BPA), nitroblue tetrazolium (NBT), oil red O (ORO) and insulin were procured from Sigma-Aldrich Co., (St. Louis, MO, USA). Dulbecco’s Modified Eagle’s Medium (DMEM), bovine serum (BS), penicillin-streptomycin (P/S), trypsin-EDTA, fetal bovine serum (FBS), horse serum (HS), and phosphate-buffered saline (PBS, pH 7.4) were obtained from Gibco (Gaithersburg, MD, USA). N-(1-naphthyl) ethylenediamine dihydrochloride was purchased from Wako Co. (Tokyo, Japan). Junsei Chemical (Tokyo, Japan) provided dimethyl sulfoxide (DMSO).

### 2.2. Sample Preparation, °Brix, pH and Lyophilization Yield

The fermented noni juice samples F1 to F4 used in this study were provided by NST Bio Co., Ltd. (Incheon, Republic of Korea), and samples F5 and F6 were obtained from Morinda, Inc. (American Fork, UT, USA). The ingredients of each noni juice sample are detailed in Table 1. For all subsequent experiments, these six samples were collectively referred to as “noni juice.” °Brix was first measured using a handheld refractometer (PAL-α Brix 0–85%, Tokyo, Japan), and the pH of each sample was determined at 25 °C employing a pH meter (pH510, EUTECH, Co., Hwaseong-si, Republic of Korea). Subsequently, each noni juice sample (90 mL) was transferred into polyethylene trays, and the initial weight of each sample was measured and documented. The samples were frozen overnight at −60 °C and then freeze-dried at a pressure of 0.005 mTorr for 7 days utilizing a freeze dryer. The freeze-dried weight was measured and recorded as the final weight. All measurements were performed in triplicate for each noni juice sample, and the results are expressed as mean ± standard deviation (SD). The lyophilization yield was determined based on Equation (1):

(1) Lyophilization yield (%) = [weight of freeze-dried powder (g)/weight of the initial powder sample (g)] × 100

### 2.3. Analysis of Bioactive Compounds Using HPLC-PDA Method

The analysis of bioactive compounds in commercial noni juice was carried out using an HPLC-PDA method modified from the method described by Choi et al. [6]. The chromatographic separation was performed on an HPLC system equipped with an SPD-M40 PDA detector (228 nm). The column was a Shiseido Capcell Pak C18 UG120 (4.6 mm × 250 mm, 5.0 μm, Tokyo, Japan) and maintained at 30 °C. The mobile phase consisted of solvent A (0.1% formic acid in DW, *v*/*v*) and solvent B (acetonitrile), with the following gradient elution: 0–5 min, 100% A; 5–30 min, linear decrease from 100 to 65% A; and 30–35 min, linear return to 100%. The total run time was 35 min, the flow rate was 1.0 mL/min, and the injection volume was 10 μL.

### 2.4. Total Phenolic, Flavonoid, and Proanthocyanidin Contents

To quantify total phenolics, each noni juice sample diluted with DW (1 mL) was combined with 10% Folin–Ciocalteu’s reagent (1 mL), followed by the addition of 2% sodium carbonate solution (1 mL). The solution was vortex-mixed and subsequently incubated in the dark for 60 min. Absorbance was determined at 750 nm using a SpectraMax i3 spectrophotometer (Molecular Devices, Sunnyvale, CA, USA). A calibration curve was established using gallic acid standards at different concentrations, and the values were reported as milligrams of gallic acid equivalents (mg GAE)/g.

For total flavonoid determination, 0.5 mL of each noni juice sample (previously diluted with DW) was mixed with 95% ethanol (1.5 mL), 10% aluminum chloride solution (0.1 mL), potassium acetate (0.1 mL), together with DW (2.8 mL). After incubation at 25 °C for 30 min, absorbance was recorded at 415 nm using a spectrophotometer. Calibration was established with quercetin standards, and results were expressed as quercetin equivalents (mg QE)/g.

Proanthocyanidin content was assessed using the 0.5% vanillin-HCl method. Each sample diluted with methanol (0.1 mL) was combined with 0.5% vanillin-HCl solution (0.5 mL) in a test tube, vortexed, and incubated in the dark for 20–30 min and the absorbance was recorded at 500 nm, and a calibration curve prepared with (+)-catechin was applied to calculate values, expressed as catechin equivalents (mg CE)/g.

### 2.5. Antioxidant Activity

#### 2.5.1. DPPH Radical Scavenging Activity

A 0.4 mM DPPH solution was prepared using anhydrous ethanol and stirred until fully dissolved. Its absorbance at 517 nm was adjusted to 1.0 ± 0.1. For the assay, 0.2 mL of each sample was combined with 0.8 mL of the DPPH solution and kept at room temperature in the dark for 10 min. Absorbance was then determined at 517 nm, and the DPPH activity was determined according to Equation (2):

(2) DPPH radical scavenging activity (%) = [1 − (A_test_/A_control_)] × 100

#### 2.5.2. ABTS Radical Scavenging Activity

A 7 mM ABTS stock solution was generated by reacting 2.45 mM potassium persulfate with ABTS (1:0.5, *v*/*v*) and storing the mixture in the dark for 16 h. The solution was then diluted until the absorbance at 734 nm reached 0.70 ± 0.02. Subsequently, each sample (10 µL) was combined with ABTS solution (1 mL), incubated for 6 min in the dark, and then monitored at 734 nm. The ABTS activity was obtained according to Equation (3):

(3) ABTS radical scavenging activity (%) = [1 − (A_test_/A_control_)] × 100

#### 2.5.3. Ferric Reducing Antioxidant Power (FRAP) Assay

To prepare the FRAP reagent, 300 mM sodium acetate buffer (pH 3.6), 10 mM TPTZ, and 20 mM FeCl_3_∙6H_2_O in a 10:1:1 (*v*/*v*) ratio. Then, the FRAP reagent (1.5 mL), each sample solution (50 μL), and DW (150 μL) were combined and incubated at 37 °C for 4 min. Finally, the absorbance of the resulting solution was then determined at 593 nm.

#### 2.5.4. Oxygen Radical Absorbance Capacity (ORAC) Assay

For the ORAC assay, 75 mM phosphate buffer at pH 7.4 was employed for solution preparation. A 144 mM AAPH solution was incubated at 37 °C for 15 min before use. Subsequently, each sample (25 µL) was then sequentially combined with 53 nM FL solution (150 µL) and 144 mM AAPH solution (25 µL) in a 96-well plate. The absorbance was immediately measured at 485 nm and 530 nm. The measurement was conducted for 90 min, with fluorescence values (f_n_) recorded at 3 min intervals. Here, f_0_ is the initial fluorescence intensity. ORAC values were derived by referencing a Trolox standard calibration curve, based on the area under the fluorescence decay curve (AUC). The AUC was determined according to Equation (4):

(4) AUC = 1 + f_1_/f_0_ + f_2_/f_0_ + f_3_/f_0_ + f_4_/f_0_ + … f_31_/f_0_

#### 2.5.5. Reducing Power Assay

To determine the reducing power assay, each sample solution (0.1 mL) was combined with 0.2 M sodium phosphate buffer (0.5 mL, pH 6.6) and 1% (*v*/*v*) potassium ferricyanide solution (0.5 mL). After incubation at 50 °C for 20 min, 0.5 mL of 10% (*w*/*v*) TCA solution was added, and centrifuged at 1790× *g* for 10 min. Subsequently, the obtained supernatant (0.5 mL) was mixed with DW (0.5 mL) and 0.1% (*w*/*v*) Iron (iii) chloride (0.1 mL). The absorbance was measured at 700 nm.

#### 2.5.6. Superoxide Dismutase (SOD)-like Activity

The SOD-like activity in each sample was determined using an OxiTecTM SOD assay kit (Biomax, Seoul, Republic of Korea) with xanthine oxidase and WST. Prepared sample solutions (20 μL each) in a 96-well plate, and then WST working solution (200 μL). Subsequently, the enzyme working solution (20 μL) was introduced into each well and mixed, followed by incubation at 37 °C for 20–30 min, and each sample’s absorbance was recorded at 450 nm. SOD activity was calculated according to Equation (5):

(5) SOD activity (Inhibition rate %) = [(OD_blank1_ − OD_blank3_) − (OD_sample_ − OD_blank2_)]/(OD_blank1_ − OD_blank3_) × 100

### 2.6. Cell Viability, Lipid Accumulation, and ROS Production in MDI- and BPA-Induced 3T3-L1 Adipocytes

3T3-L1 cells (ATCC, #CL-173; Manassas, VA, USA) were maintained in DMEM (10% BS, 3.7 g/L sodium bicarbonate, and 1% P/S) at 37 °C in a 5% CO_2_. Differentiation was initiated two days after confluence, when cells were transferred to 10% FBS medium containing adipogenic inducers (MDI), which consisted of 0.5 mM IBMX, 1 μM DEX, and 1 μg/mL insulin. Samples were treated at 200 and 400 μg/mL. Then, the 10% FBS medium with 1.0 μg/mL insulin was replaced every 48 h until differentiation reached day 8, when adipocyte proliferation was evaluated using the 2,3-bis(2-methoxy-4-nitro-5-sulfophenyl)-2H-tet-razolium-5-carboxanilide (XTT) assay (WelGene, Seoul, Republic of Korea). A mixture of N-methyl dibenzopyrazine methyl sulfate (PMS) and XTT was dispensed into 96-well plates, then incubation at 37 °C in 5% CO_2_ for 4 h, and absorbance was determined at 450 and 690 nm.

To assess the degree of lipid accumulation, ORO staining was conducted on day 8 on cells treated with noni juice extract (400 μg/mL). Cells were fixed using a 10% formalin solution for one hour, washed with 60% isopropanol (500 μL), air-dried, and subsequently stained with ORO working solution for 30 min. Lipid-bound ORO was subsequently extracted using 100% isopropanol, and the absorbance was quantified at 490 nm.

Reactive oxygen species (ROS) production was analyzed using the NBT assay. On day 8, cells were incubated with a 0.2% NBT working solution in a CO_2_ incubator for 90 min. The dark blue formazan product was dissolved using 300 μL of DMSO and 1 N KOH mixture (7:3, *v*/*v*), followed by dilution with an equal volume of DW. Absorbance was then measured spectrophotometrically at 570 nm.

In the BPA-induced anti-obesogenic assay, cells were cultured for 6 days in 20 μM BPA before differentiation. On day 0, differentiation was induced with DMEM containing 1% P/S, 1.0 μg/mL insulin, 0.5 mM IBMX, 20 μM BPA, and 10% FBS. After two days, the medium was changed to fresh DMEM with 20 μM BPA, 1% P/S, 10% FBS, and 1.0 μg/mL insulin, and subsequently renewed every two days. Cells were cultured until day 10 of differentiation, after which cell viability, lipid accumulation, and ROS generation were evaluated using the above-described methods to assess the anti-obesogenic effects of each noni juice sample.

### 2.7. Evaluation of the Anti-Muscle Atrophy Effects in C2C12 Cells

C2C12 myoblasts (ATCC, Manassas, VA, USA) were cultured in DMEM containing 10% FBS, 3.7 g/L sodium bicarbonate, and 1% P/S at 37 °C in a 5% CO_2_. The medium was replaced every two days until confluence. Then, the cells were grown in DMEM containing 2% HS and 1% P/S to induce differentiation. On day 5 of differentiation, noni juice samples at 200 and 400 µg/mL along with 200 µM DEX for a duration of 24 h. Then, cell viability was determined utilizing the same XTT assay. For Jenner-Giemsa staining, cells were initially fixed in 100% methanol for 5 min, and then air-dried for 10 min. Then, Jenner staining solution (300 µL) was applied to each well for 5 min, followed by two washes with DW. Subsequently, an equal volume of the Giemsa staining solution, stained for 10 min, and the wells were again rinsed twice with DW.

Images were captured using an inverted microscope (CKX41SF, Tokyo, Japan). Five random images were obtained at 200× magnification. The number of myotubes containing at least three nuclei was quantified using the ImageJ software (version 1.53k). The fusion index was subsequently determined according to Equation (6):

(6) Fusion index (%) = (number of nuclei in myotubes/total number of nuclei) × 100

### 2.8. Measurements of Immunoenhancing and Anti-Inflammatory Effects in RAW 264.7 Cells

RAW 264.7 macrophages (ATCC, TIB-71, Manassas, VA, USA) were maintained in DMEM with 10% FBS, 1% P/S, and 3.7 g/L sodium bicarbonate at 37 °C in 5% CO_2_ until confluence. Subsequently, the cells were treated with each noni juice sample at the concentrations of 200 and 400 μg/mL for 24 h. Cell viability was assessed using the same XTT assay.

After 24 h of treatment with each concentration of noni juice samples, Griess reagent solution (100 μL) was mixed with each sample culture medium (100 μL) and incubated for 10 min. The Griess reagent solution was prepared by mixing 0.1% N-(1-naphthyl) ethylenediamine dihydrochloride in DW and 1% sulfanilamide in 5% phosphoric acid (H_3_PO_4_) in the 1:1 ratio. The absorbance at 550 nm was determined to evaluate nitric oxide (NO) production.

### 2.9. Measurement of Protective Effects in UVB-Induced HDF Cells

Human dermal fibroblasts (HDF) (ATCC, PCS-201-012, Manassas, VA, USA) were maintained in low-glucose DMEM with 10% FBS and 1% P/S at 37 °C in 5% CO_2_. Cells were maintained with medium changes every 3 days, and cells were subcultured at 80% confluence. For ultraviolet B (UVB) exposure, cells were irradiated with a Bio-Link Crosslinker (Vilber Lourmat, Cedex, Collégien, France) at an intensity of 100 mJ/cm^2^. XTT assay was used to determine cell viability. HDF cells were plated at 1.2 × 10^5^ cells/well in 6-well plates. Cells were incubated for 24 h, washed twice with PBS and then exposed to 10 mJ/cm^2^ UVB irradiation with a thin layer of PBS. Following UVB exposure, the cells were treated with the noni juice sample, and matrix metalloproteinase-1 (MMP-1) activity was measured by the Human Pro MMP-1 Quantikine ELISA Kit (R&D Systems, Minneapolis, MN, USA).

### 2.10. Statistical Analysis

Results are expressed as mean ± SD from triplicate experiments. Statistical analysis was performed using one-way ANOVA followed by Duncan’s test, with significance set at *p* < 0.05 (SAS Institute, Inc., Cary, NC, USA). All results were presented using box plots to visually represent the data distribution and statistical differences.

## 3. Results

### 3.1. Quantification of Bioactive Compounds in Noni Juice by HPLC-PDA

As the primary objective of this study was to assess the overall functional potential of commercially available noni juice products in Korea, the analytical results of the six products were integrated and expressed as mean values.

**Figure 1:**
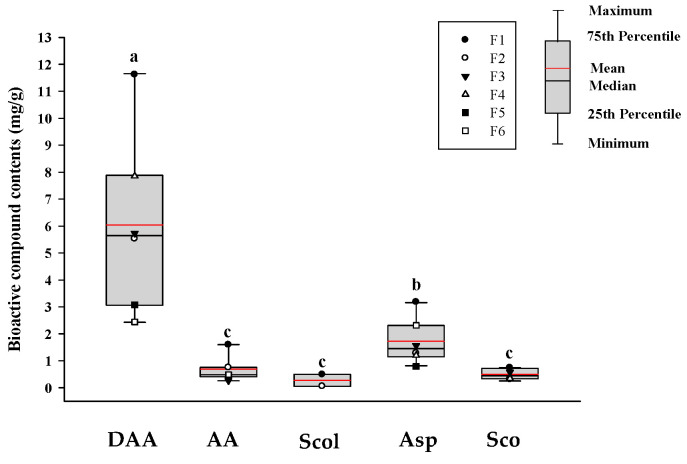
HPLC-PDA determination of bioactive compounds in commercial noni juice. The black and red lines show the median and mean values, respectively, and the black dots represent the outliers. According to Duncan’s multiple range test, different lowercase letters indicate statistically significant differences at *p* < 0.05. DAA: deacetylasperulosidic acid; AA: asperulosidic acid; Scol: scopolin; Asp: asperuloside; Sco: scopoletin.

As shown in Figure 1, the contents of five bioactive compounds in commercial noni juice samples were quantitatively analyzed using HPLC-PDA (Shimadzu LC system, LC-40B XR, Shimadzu Co., Ltd., Kyoto, Japan). These compounds included DAA, AA, Scol, Asp, and Sco. The findings indicated that the average content of DAA was 6.04 ± 3.07 mg/g, which was markedly greater than that of the other compounds, suggesting that it may be the principal iridoid compound in noni juice. The average contents of the remaining compounds were 1.73 ± 0.80 mg/g for Asp, 0.70 ± 0.47 mg/g for AA, 0.50 ± 0.18 mg/g for Sco, and 0.28 ± 0.22 mg/g for Scol, with Scol exhibiting the lowest average content. These findings provide a comprehensive overview of the distribution of major bioactive compounds in commercial noni juices.

### 3.2. °Brix, pH, Lyophilization Yield, and Content of Total Phenolics, Flavonoids, and Proanthocyanidins in Noni Juice

As shown in Table 2, the lyophilization yield, physicochemical properties (°Brix and pH values), and the contents of flavonoids, phenolics, and proanthocyanidins in six commercial noni juice were systematically evaluated. The results showed that the average °Brix and pH values were 15.16 ± 4.08% and 3.63 ± 0.29, respectively, indicating a moderate sugar content and relatively low acidity. The lyophilization yield was 15.42 ± 4.62%, reflecting high retention of solid constituents. In addition, the contents of phenolics and flavonoids were 6.39 ± 1.45 mg GAE/g and 1.47 ± 0.31 mg QE/g, respectively, while the proanthocyanidin content was the highest, reaching 8.64 ± 6.20 mg CE/g. These findings suggested that commercial noni juice contains substantial amounts of phenolic compounds, which are likely to play a key role in its antioxidant potential and related physiological effects.

### 3.3. Effect of Noni Juice on Antioxidant Activity

This study conducted a comprehensive evaluation of the antioxidant properties of commercial noni juice through multiple in vitro assays, including ABTS and DPPH free radical scavenging assays, reducing power, FRAP activity, and SOD-like activity. As shown in Table 3 the antioxidant activities of the six commercial noni juice samples exhibited some variation, but the overall trends were consistent. The ABTS scavenging activity was the highest, averaging 55.91 ± 2.62%, followed by DPPH scavenging activity at 44.03 ± 14.88%, and SOD-like activity at 4.10 ± 0.92 Unit/mg. In addition, the ORAC value was 1.24 ± 0.50 µM TE/mg, further reflecting its strong potential to scavenge oxygen radicals. FRAP and reducing power were 0.23 ± 0.62 and 0.16 ± 0.05, respectively. Collectively, these findings indicate that noni juice has significant antioxidant activity.

### 3.4. Effects of Noni Juice on Anti-Obesity and Anti-Obesogenic Activities

**Figure 2:**
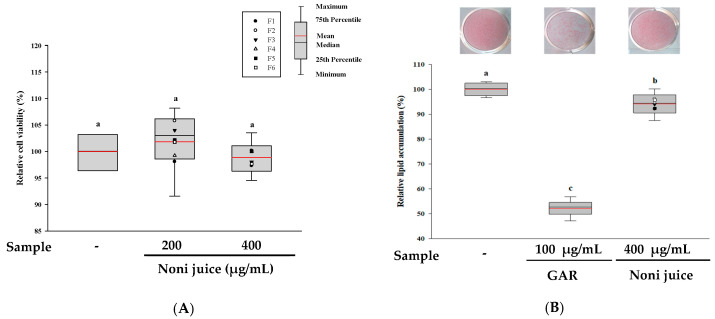

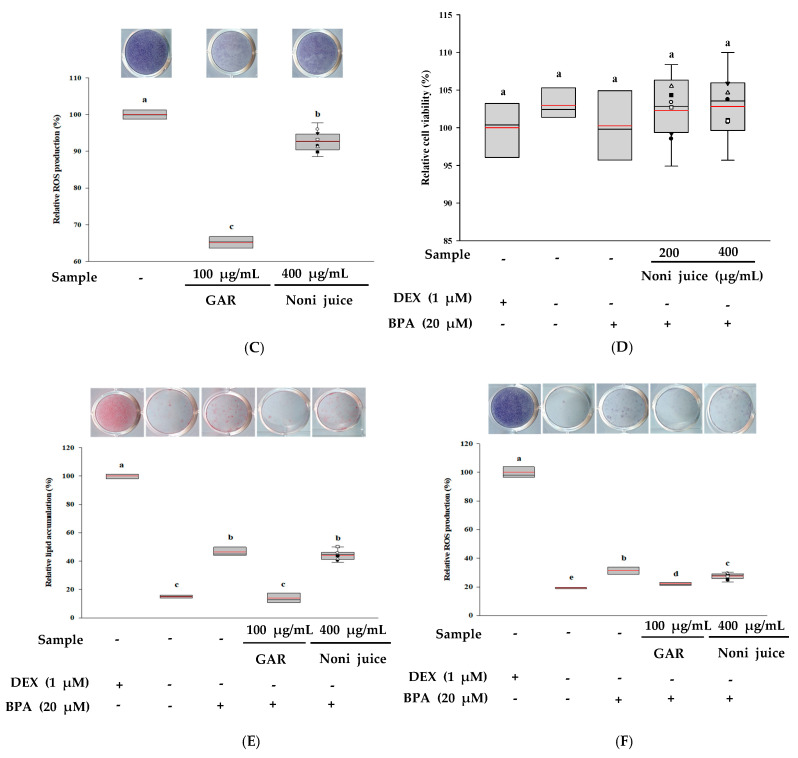
Effects of noni juice samples on cell viability, lipid accumulation, and ROS production in MDI-treated (**A**–**C**) and BPA-induced adipocytes (**D**–**F**). (**A**,**D**) Cell viability was using the XTT assay; (**B**,**E**) lipid accumulation assessed through ORO staining; (**C**,**F**) relative ROS production quantified by the NBT assay. The black and red lines show the median and mean values, respectively, and the black dots represent the outliers. Different lowercase letters indicate statistically significant differences at *p* < 0.05. GAR: garcinia cambogia; BPA: bisphenol A; DEX: dexamethasone.

First, the cytotoxicity of noni juice in 3T3-L1 adipocytes was evaluated by the XTT assay. As shown in Figure 2A,D, treatment with noni juice at 200 and 400 µg/mL did not significantly reduce cell viability, indicating no cytotoxicity within the experimental concentration range. In addition, the lipid accumulation levels were assessed by ORO staining, and garcinia cambogia (GAR), which has anti-obesity activity, employed as the positive control [22]. The results showed that treatment with 400 µg/mL noni juice reduced lipid accumulation by approximately 5.92% relative to the control group (Figure 2B). In the BPA-induced adipogenesis model, noni juice at 400 µg/mL slightly decreased lipid accumulation by approximately 2.16% compared to the BPA group. This reduction was statistically significant (Figure 2E). To further evaluate cellular oxidative metabolism, an NBT assay was performed [23]. As shown in Figure 2C, treatment with noni juice markedly decreased dark-blue formazan formation, which corresponded to a 7.23% reduction in ROS production compared with the control group. Moreover, under BPA-induced conditions, 400 µg/mL of noni juice reduced ROS production by 3.85% relative to the BPA group (Figure 2F). These results indicate that noni juice exhibits moderate anti-obesity activity by inhibiting lipid accumulation and effectively alleviating oxidative stress in adipocyte models.

### 3.5. Anti-Muscle Atrophy Effects of Noni Juice in C2C12 Myotubes

**Figure 3:**
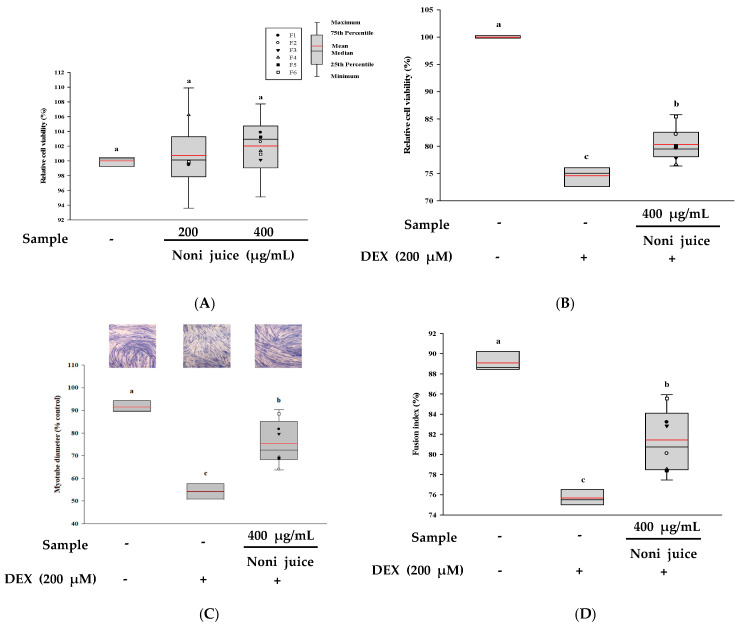
Effects of noni juice samples on DEX-induced C2C12 myotubes. (**A**) Cytotoxicity analysis; (**B**) assessment of cytoprotective effects; (**C**) measurement of myotube diameter, and (**D**) calculation of fusion index. The black and red lines show the median and mean values, and the black dots represent the outliers. Different lowercase letters indicate statistically significant differences at *p* < 0.05. DEX: dexamethasone.

The effects of noni juice at 200 and 400 µg/mL on C2C12 myotube viability were assessed using the XTT assay. Figure 3A shows that none of the tested concentrations caused cytotoxicity. To further evaluate the cytoprotective effects of noni juice, C2C12 myotubes were exposed with 200 µM DEX, and the outcome is presented in Figure 3B. Compared with the group using only DEX, the cell viability of the experimental group treated with 400 µg/mL noni juice was significantly increased. In addition, the myotube cells were stained and analyzed using the Jenner-Giemsa staining. Figure 3C shows that the myotube diameter in the DEX group was significantly reduced by 37.24% relative to that in the control group. However, co-treatment with 400 µg/mL noni juice effectively restored the myotube diameter, indicating its ability to attenuate DEX-induced myotube atrophy. Furthermore, the fusion index, which reflects the maturation and differentiation of myotubes, was significantly decreased by the DEX treatment but was markedly restored to 81.44% following co-treatment with noni juice (Figure 3D). These results demonstrated that noni juice effectively improved DEX-induced muscle atrophy by enhancing cell viability, preserving myotube morphology, and promoting myotube fusion.

### 3.6. Evaluation of Immunoenhancing and Anti-Inflammatory Effects of Noni Juice in RAW 264.7 Cells

**Figure 4:**
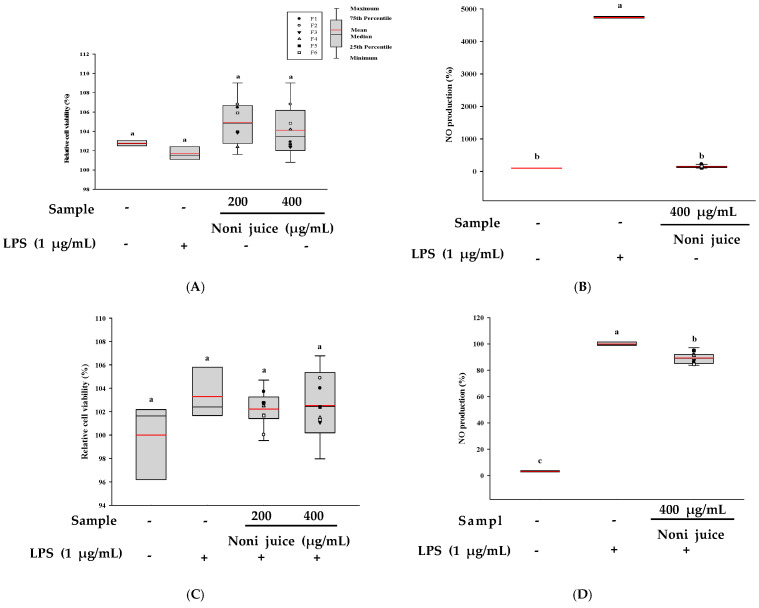
Effects of noni juice on cell viability and NO production in RAW 264.7 cells. (**A**) Cell viability under immunoenhancing experimental conditions; (**B**) NO production under immunoenhancing experimental conditions; (**C**) cell viability under anti-inflammatory experimental conditions, and (**D**) NO production under anti-inflammatory experimental conditions. The black and red lines show the median and mean values, and the black dots represent the outliers. Different lowercase letters indicate statistically significant differences at *p* < 0.05. LPS: lipopolysaccharides.

The impact of noni juice on RAW 264.7 cells under immunoenhancing and anti-inflammatory conditions were assessed using the XTT assay. As shown in Figure 4A,C, treatment with noni juice at 200 and 400 µg/mL did not result in any notable cytotoxicity relative to the control groups. Furthermore, to explore the immunomodulatory effects of noni juice, NO production in the cells was determined by the Griess assay. The results revealed that under immunoenhancing experimental conditions, the LPS-treated group showed the highest level of NO production. However, the NO production level in the group treated with 400 µg/mL noni juice was close to that in the control group, showing no increasing trend, indicating that noni juice did not exert a significant immunoenhancing effect (Figure 4B). Conversely, under anti-inflammatory experimental conditions, the LPS-treated group showed significantly increased NO production, whereas treatment with 400 µg/mL noni juice under LPS-induced conditions reduced NO production by approximately 10.64% compared with the LPS group (Figure 4D). Overall, the data imply that noni juice exhibits anti-inflammatory properties by reducing NO generation.

### 3.7. Cytoprotective Properties of Noni Juice on HDF Cells Exposed to UVB Irradiation

**Figure 5:**
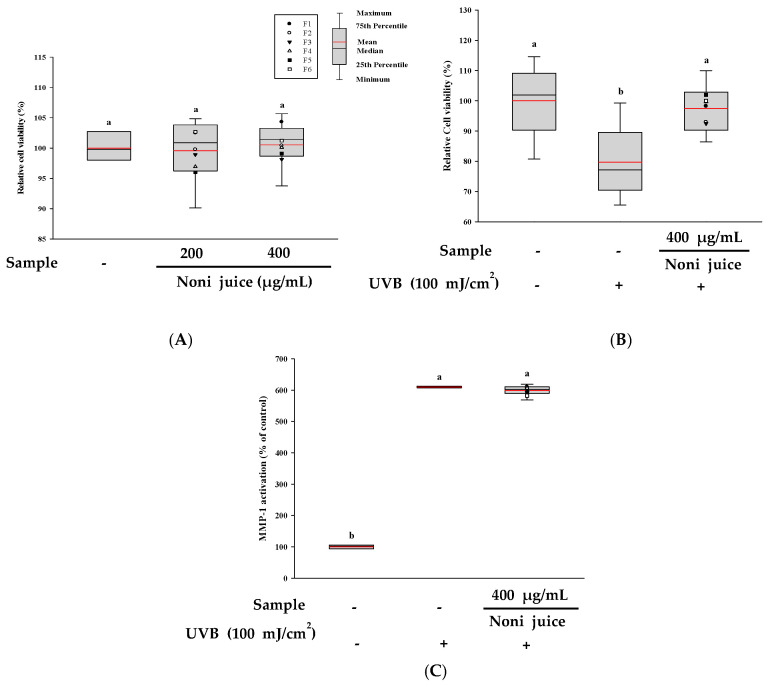
Cytoprotective effects of noni juice against UVB-induced damage in HDF cells. (**A**) Cell viability following treatment with noni juice alone; (**B**) cell viability following UVB exposure with noni juice treatment, and (**C**) MMP-1 activity levels as an indicator of extracellular matrix degradation following UVB exposure. The black and red lines show the median and mean values, and the black dots represent the outliers. Different lowercase letters indicate statistically significant differences at *p* < 0.05. UVB: ultraviolet B radiation; MMP-1: matrix metalloproteinase-1.

To assess the cytoprotective properties of noni juice against UVB-induced damage in HDF cells, the cells were first treated with 200 and 400 µg/mL of noni juice and then subjected to UVB irradiation. As shown in Figure 5A, treatment with noni juice alone did not induce cytotoxicity at either concentration, and cell viability remained comparable to that of the control group. After UVB exposure (100 mJ/cm^2^), the cell viability decreased significantly to 79.71%. However, treatment with 400 µg/mL noni juice restored viability to 97.52% (Figure 5B). To further explore the potential skin protection mechanism, the activity of MMP-1 was assessed. As shown in Figure 5C, UVB irradiation (10 mJ/cm^2^) induced significant secretion of MMP-1 in HDF. However, treatment with noni juice did not significantly reduce MMP-1 activity, indicating that noni juice has no significant inhibitory effect on the UVB-induced MMP-1 activity. These results suggest that although noni juice consumption may have a specific cytoprotective effect, the anti-photoaging effect may not be limited to noni juice consumption alone, and that direct application of noni juice extract to the skin may also have a significant anti-photoaging effect.

## 4. Discussion

Research has shown that noni (*Morinda citrifolia* L.) has traditionally been used as a medicinal plant and has garnered significant attention for its nutritional composition and diverse physiological functions. However, since the taste and flavor of fresh noni fruit are not suitable for direct consumption, they are made into juice and consumed. Recently, noni juice made through fermentation has become a new popular food [2,24]. This study demonstrates the potential of noni juice as a functional beverage by analyzing the bioactive compounds and physiological activities of commercially available noni juice samples. HPLC-PDA analysis results revealed that noni juice was rich in iridoid compounds, particularly DAA, which is a major phytochemical in noni juice. Recent metabolomic research further reported that fermentation can alter the iridoid composition of noni juice. Fermented samples showed higher levels of DAA and monotropein, whereas non-fermented ones contained more AA and rutin [4]. These findings together suggest that both DAA and other iridoid derivatives formed or transformed during fermentation may also contribute to the antioxidant potential of noni juice. In addition, previous studies have reported that these iridoids exhibit significant antioxidant and anti-inflammatory properties, which may be the key factors contributing to the health benefits of noni juice [25]. In the present study, noni juice exhibited a relatively high level of phenolic and flavonoid compounds, which may further enhance its antioxidant activity. Consistent with previous studies, these phytochemicals are considered the major reason for the strong antioxidant activity of noni juice [26,27]. Compared to other commercial fruit juices reported in the literature, noni juice exhibited superior antioxidant capacity. For example, the DPPH activity of noni juice (44.03 ± 14.88%) was higher than that of the commonly consumed juices such as blueberry juice (20.6 ± 1.4%), orange juice (12.7 ± 1.0%), and apple juice (11.8 ± 1.9%), suggesting strong free radical scavenging effects [20]. This difference may be attributed to the unique phytochemical composition of noni juice, particularly its richness in key bioactive compounds such as DAA and Sco, which are uncommon in other fruit juices that are mainly abundant in vitamin C or anthocyanins. In addition, partial fermentation processes used in certain commercial noni juice products may promote the release and bioavailability of antioxidant constituents through the hydrolysis of glycosidic bonds, thereby enhancing their radical scavenging potential [16,28]. Moreover, a recent comparative study reported that fruit juices such as wild rose and chokeberry generally exhibited higher antioxidant capacities, whereas noni juice showed moderate activity. Its antimicrobial potential also varied depending on the phenolic composition. These differences may result from variations in cultivar and origin, processing methods, and juice formulation rather than from a single fruit source itself [29].

Obesity is a chronic and complex metabolic disease that typically results from excessive dietary intake and genetic factors, leading to excessive fat accumulation in the body [30]. In the present study, noni juice exhibited a certain degree of anti-obesity effects by reducing lipid accumulation and intracellular ROS production, indicating its potential benefits in alleviating oxidative stress related to adipogenesis. Previous research has shown that polyphenolic compounds play important roles in regulating adipogenesis and promoting lipolysis, and are key compounds for the treatment of obesity and other diseases [31,32]. Therefore, the high total phenolic content observed in this research suggests that the anti-obesity effects of noni juice are closely related to its abundant polyphenolic compounds. The reduction in lipid accumulation may also be influenced by iridoid glycosides, such as DAA and Asp, which have been reported to activate AMPK and inhibit adipogenesis by downregulating the master transcription factors PPARγ and C/EBPα, as well as their lipogenic targets, including FAS [33]. In addition, animal studies by Nishioka and Nerurkar [34], and Shoeb et al. [35] confirmed that noni juice has multiple metabolic regulatory effects, including anti-obesity, hypoglycemic, and hypolipidemic effects. Beyond these direct effects on adipocytes, noni phenolics have also been shown to mitigate HFD-induced weight gain through modulation of gut microbiota and reduction in intestinal inflammation, suggesting that attenuation of intestinal inflammation may contribute to the anti-obesity effects of noni products [36].

Skeletal muscle plays a critical role in maintaining physical mobility and regulating systemic metabolism. Reduced myofiber contractility and muscle mass loss are among the primary causes of muscle atrophy [37]. In this study, noni juice demonstrated protective effects in a dexamethasone-induced C2C12 myotube atrophy model. The increase in myotube diameter and fusion index further indicated its potential role in promoting muscle health. Previous studies have reported that noni juice and its bioactive components have beneficial effects on bone metabolism. Shirwaikar et al. [38] observed that noni juice extract effectively prevented bone loss in ovariectomized rats. In addition, other studies have suggested that noni juice exhibits anti-arthritic activity, which could be linked to its elevated content of flavonoid and phenolic compounds [39]. These findings suggest that noni juice exerts dual protective effects on bone and muscle tissues, possibly through its antioxidant or anti-inflammatory properties.

Inflammation is an immune defense mechanism activated in response to infection or tissue injury, and is characterized by the activation and recruitment of macrophages, along with the release of various inflammatory mediators [40]. LPS, a hallmark of Gram-negative bacteria, is often employed to induce inflammation. It significantly promotes the expression of proinflammatory cytokines, thereby inducing inflammatory responses [41]. NO is an important mediator of immune activation and inflammatory responses, and its excessive production is often associated with the induction of inflammation [42,43]. According to the research by Choi et al. [44], iridoid compounds such as DAA and Asp can inhibit the activation of the NF-κB signaling pathway, thereby reducing the expression of proinflammatory cytokines, including TNF-α, IL-6, and IL-1β. In addition, previous studies have indicated that the polyphenolic compounds abundant in noni juice also have anti-inflammatory properties [27]. Thus, the anti-inflammatory effects observed in this study may be closely associated with the high content of phenolics and iridoids in noni juice samples, further demonstrating the potential of noni juice as an anti-inflammatory functional beverage.

UVB irradiation reduces skin elasticity and promotes wrinkle formation by inducing oxidative stress and inflammatory processes [45]. MMP-1 is an enzyme involved in breaking down the extracellular matrix (ECM), particularly collagen. Its expression is upregulated upon UVB exposure, thereby accelerating collagen degradation and causing pigmentation, and skin aging [46,47,48]. In vivo, in vitro, and clinical studies by West [49] demonstrated that noni juice inhibits UVB-induced MMP-1 production and promotes collagen synthesis, thereby improving skin health. However, while commercially available noni juice demonstrated specific cytoprotective effects, it did not significantly reduce MMP-1 levels, suggesting that direct skin application, rather than ingestion only, is necessary for a significant anti-aging effect. The differences between specific cell protection and photoaging prevention may stem from differences in raw material sources, processing methods, and application methods (ingestion versus direct skin application). Therefore, more studies are required to clarify its mechanism of action.

These findings suggest that noni juice exerts multiple physiological effects, particularly in antioxidant, anti-muscle atrophy, anti-obesity, and anti-inflammatory activities. The results highlight its potential as a valuable functional food and beverage for health promotion. However, further studies are needed to elucidate its underlying mechanisms and to explore its wider applications.

## 5. Conclusions

Overall, this study systematically investigated the major bioactive compounds and various physiological activities of commercial noni juice products in Korea. HPLC-PDA revealed that noni juice is rich in polyphenolic compounds (6.39 ± 1.45 mg GAE/g) and iridoids, including DAA (6.04 ± 3.07 mg/g) and Asp (1.73 ± 0.80 mg/g), which are likely to be key contributors to its antioxidant activity. In vitro experiments further demonstrated that noni juice reduced lipid accumulation and ROS production and exerted a protective effect in a dexamethasone-induced C2C12 myotube atrophy model. Additionally, noni juice exhibits anti-inflammatory effects by regulating NO production. A comparative analysis of the results obtained in this study for noni juice and the results obtained in previous studies of commercial fruit juices (such as apple, blueberry, and orange juices) suggests that noni juice exhibits relatively high antioxidant activity and functional potential, possibly due to its mixed juice composition and synergistic effects among bioactive compounds. These findings provide scientific evidence supporting the development of noni juice as a functional food and healthy beverage and highlight its promising prospects for commercial applications.

## Figures and Tables

**Table 1:** Main ingredients as indicated on the labels of commercial noni juice samples used in this study.

Sample	Main Ingredients	Manufacturer ^1^
F1	Fermented and ripened noni juice.	NST Bio
F2	Fermented and ripened noni juice, coconut sugar, calamondin juice.	NST Bio
F3	Fermented and ripened noni juice, fructooligosaccharides, aronia juice concentrate, fermented noni concentrate, bilberry concentrate.	NST Bio
F4	Fermented and ripened noni juice, fructooligosaccharides, seven berry concentrate mix, fermented noni concentrate.	NST Bio
F5	Noni fruit puree, blueberry juice concentrate, grape juice concentrate.	Morinda
F6	Noni fruit puree, cornelian cherry puree, cornelian cherry reconstituted juice, olive leaf extract.	Morinda

^1^ Manufacturer names were coded. F1–F4: fermented noni-based commercial juices. F5–F6: noni-puree-based commercial juice.

**Table 2:** Mean values of total soluble solid (°Brix), pH value, yields, and bioactive compounds of noni juice.

Noni Juice	°Brix (%)	pH	Lyophilization Yield (%)	Total Phenolic Content (mg GAE ^1^/g)	Total Flavonoid Content (mg QE ^2^/g)	Total Proanthocyanidin Content (mg CE ^3^/g)
F1	8.80 ± 0.08 ^e^	3.88 ± 0.01 ^b^	7.74 ± 0.05 ^d^	7.50 ± 0.01 ^b^	1.28 ± 0.04 ^c^	11.96 ± 0.34 ^b^
F2	15.78 ± 0.09 ^c^	3.97 ± 0.01 ^a^	16.11 ± 0.04 ^b^	4.58 ± 0.07 ^f^	1.09 ± 0.03 ^d^	3.95 ± 0.54 ^d^
F3	19.57 ± 0.19 ^b^	3.77 ± 0.01 ^c^	20.26 ± 0.29 ^a^	7.38 ± 0.02 ^c^	1.60 ± 0.08 ^b^	20.61 ± 0.33 ^a^
F4	15.80 ± 0.00 ^c^	3.65 ± 0.00 ^d^	16.30 ± 0.41 ^b^	5.24 ± 0.01 ^d^	1.24 ± 0.06 ^c^	6.98 ± 0.05 ^c^
F5	11.10 ± 0.00 ^d^	3.22 ± 0.00 ^f^	11.38 ± 0.23 ^c^	5.16 ± 0.01 ^e^	1.63 ± 0.05 ^b^	6.54 ± 0.05 ^c^
F6	19.9 ± 0.00 ^a^	3.28 ± 0.01 ^e^	20.70 ± 0.36 ^a^	8.45 ± 0.03 ^a^	2.00 ± 0.06 ^a^	1.80 ± 0.18 ^e^
Mean ± SD	15.16 ± 4.08	3.63 ± 0.29	15.42 ± 4.62	6.39 ± 1.45	1.47 ± 0.31	8.64 ± 6.20

^1^ GAE: gallic acid equivalent. ^2^ QE: quercetin equivalent. ^3^ CE: catechin equivalent. ^a–f^: different lowercase letters within the same column indicate statistical differences at *p* < 0.05 by Duncan’s multiple range test.

**Table 3:** Mean values of antioxidant activities of noni juice.

Noni Juice	DPPH Radical Scavenging (%)	ABTS Radical Scavenging (%)	FRAP Activity (O.D. 593 nm)	Reducing Power (O.D. 700 nm)	SOD-Like Activity (Unit/mg)	ORAC Value (μM TE ^1^/mg)
F1	45.96 ± 0.61 ^c^	57.10 ± 0.10 ^c^	0.24 ± 0.00 ^c^	0.17 ± 0.00 ^b^	4.01 ± 0.01 ^d^	1.13 ± 0.06 ^c^
F2	26.44 ± 0.26 ^f^	53.20 ± 0.76 ^e^	0.16 ± 0.00 ^f^	0.12 ± 0.00 ^e^	2.23 ± 0.03 ^e^	0.79 ± 0.00 ^d^
F3	50.89 ± 0.07 ^b^	58.27 ± 0.52 ^b^	0.27 ± 0.00 ^b^	0.17 ± 0.00 ^b^	4.83 ± 0.07 ^b^	2.18 ± 0.13 ^a^
F4	33.25 ± 0.41 ^e^	52.99 ± 0.38 ^e^	0.18 ± 0.00 ^e^	0.12 ± 0.00 ^d^	4.04 ± 0.05 ^d^	0.71 ± 0.09 ^d^
F5	35.64 ± 1.76 ^d^	54.21 ± 0.18 ^d^	0.20 ± 0.00 ^d^	0.13 ± 0.00 ^c^	4.43 ± 0.01 ^c^	1.16 ± 0.02 ^c^
F6	71.98 ± 0.42 ^a^	59.71 ± 0.10 ^a^	0.34 ± 0.00 ^a^	0.25 ± 0.00 ^a^	5.05 ± 0.04 ^a^	1.49 ± 0.08 ^b^
Mean ± SD	44.03 ± 14.88	55.91 ± 2.62	0.23 ± 0.62	0.16 ± 0.05	4.10 ± 0.92	1.24 ± 0.50

^1^ TE: Trolox equivalents. ^a–f^: different lowercase letters within the same column indicate statistical differences at *p* < 0.05 by Duncan’s multiple range test.

## Data Availability

The original contributions presented in the study are included in the article, further inquiries can be directed to the corresponding author.

## Abbreviations

The following abbreviations are used in this manuscript:

GAE	Gallic acid equivalents
CE	Catechin equivalents
DPPH	1,1-diphenyl-2-picrylhydrazyl
ABTS	2,2′-azino-bis (3-ethylbenzothiazoline-6-sulfonic acid) diammonium salt
ROS	R eactive oxygen species
NO	Nitric oxide
DAA	Deacetylasperulosidic acid
Asp	Asperuloside
AA	Asperulosidic acid
Sco	Scopoletin
HPLC	High-performance liquid chromatography
PDA	Photodiode array
LPS	Lipopolysaccharides
IBMX	3-isobutyl-1-methylxanthine
BPA	Bisphenol A
DEX	Dexamethasone
NBT	Nitroblue tetrazolium
ORO	Oil red O
QE	Quercetin equivalents
FRAP	Ferric-reducing antioxidant power
ORAC	Oxygen radical absorbance capacity
SOD	Superoxide dismutase
UVB	Ultraviolet B
MMP-1	Matrix metalloproteinase-1
GAR	Garcinia cambogia
ECM	Extracellular matrix
